# Surgical Site Infection After Stoma Reversal: A Comparison Between Linear and Purse-String Closure

**DOI:** 10.7759/cureus.50057

**Published:** 2023-12-06

**Authors:** Muhammad Awais Khan, Khurram Niaz, Shahzeb Asghar, Maaz A Yusufi, Mohtamam Nazir, Syed Muhammad Ali, Aryan Ahmed, Akeel Ahamed Salahudeen, Talha Kareem

**Affiliations:** 1 Accident and Emergency, Frimley Health National Health Service (NHS) Foundation Trust, Surrey, GBR; 2 General Surgery, Sheikh Zayed Medical College and Hospital, Rahim Yar Khan, PAK; 3 General Surgery, Multan Medical and Dental College, Multan, PAK; 4 General Surgery, University Hospitals Dorset National Health Service (NHS) Foundation Trust, Dorset, GBR; 5 Surgery, Weill Cornell Medical School, Doha, QAT; 6 Acute Care Surgery, Hamad General Hospital, Doha, QAT; 7 Surgery, Nishtar Hospital, Multan, PAK; 8 General Surgery, Nishtar Hospital, Multan, PAK

**Keywords:** purse-string closure, colostomy, ileostomy, stoma formation, linear skin closure

## Abstract

Introduction: Intestinal stomas are utilized for both benign and malignant conditions of the intestine to mitigate the risk of anastomotic leakage and re-exploration. However, stomas are associated with various complications, such as stoma necrosis, peri-stomal irritation, parastomal hernia, bleeding, bowel obstruction, and electrolyte abnormalities. Surgical site infection (SSI) is a significant source of morbidity following stoma reversal, leading to increased patient morbidity. The conventional method of stoma reversal involves closing the skin with non-absorbable sutures in a linear fashion, which is known as linear skin closure (LSC). Recently, a new method of skin closure using purse-string approximation (PSA) has been advocated, which allows healing by secondary intention. The rationale for this study is to compare the SSI associated with LSC and PSA after stoma reversal.

Objective: This study aims to compare the frequency of SSI between LSC and PSA in stoma reversal.

Materials and methods: The study was conducted at the Department of General Surgery, Shifa International Hospitals Ltd. (SIH), Islamabad, Pakistan. The study is a randomized controlled clinical trial carried out between the 14^th^ of March 2021 and the 22^nd^ of November 2022. The sampling technique was non-probability consecutive random sampling. The sample size was calculated using the WHO sample size calculator by using the hypothesis test for two population proportions. The minimum sample size in each group was 40 patients. The total sample size was 80 patients.

Results: The overall frequency of SSI in all the patients was 18/80 (22.5%). The frequency of SSI in Group 1 (LSC) was 6/40 (15.0%), and in Group 2 (PSA), it was 12/40 (30.0%). The frequency of SSI in Group 2 (PSA) was twice as high as in Group 1 (LSC); however, the p-value was calculated to be 0.108. Therefore, this difference was statistically insignificant.

Conclusions: While PSA has exhibited promise in reducing SSI rates and enhancing aesthetic outcomes and patient satisfaction, there is still enough data favoring LSC. Moreover, insufficient data is available for our population to make a definitive statement. Consequently, further research on this topic is warranted, preferably involving larger sample sizes and multicenter randomized controlled trials, to establish which technique is superior in SSI reduction.

## Introduction

The term "stoma" comes from the Greek word "mouth or opening." It refers to an artificial opening of the bowel on the skin, which can be temporary or permanent. A German surgeon, Baum, made the first stoma in 1879 to bypass a stenotic lesion in the colon. About 150,000 stomas are created annually in the US, with equal numbers of ileostomies and colostomies [1,2]. Stomas are used for both benign and malignant colorectal conditions, and they can reduce the risk of anastomotic leakage and reoperation [3,4]. The main reasons for making a stoma are to divert the gut contents, protect the distal anastomosis, decompress the bowel, or a combination [5]. Stomas can cause complications such as stoma necrosis, peri-stomal irritation, parastomal hernia, bleeding, bowel obstruction, and electrolyte abnormalities [6,7]. Preferably, stoma reversal is done as early as possible to restore bowel continuity and prevent these complications.

Closure of a stoma is usually an elective procedure, and it is classified as a clean-contaminated surgery, which means that there is a greater risk of surgical site infection (SSI) in and around the stoma site [8]. Closure of the skin with non-absorbable sutures in a linear fashion after stoma closure has been the traditional method. In 1997, Banerjee proposed a new method of skin closure after stoma reversal using purse-string approximation (PSA), which allows healing by secondary intention [9]. Despite this novel method, linear skin closure (LSC) is still widely used.

SSI is a major cause of morbidity after stoma reversal. A study conducted in 2021 showed that the SSI rate after stoma reversal was as high as 29% [10]. With LSC, data has shown the infection rate varying from 2% to an alarming 40% [11,12]. In the case of PSA, the infection rate has been lower and as good as 2% vs. 15% compared to LSC in a study published in 2014 [13]. Therefore, reducing the SSI rate should be of tantamount importance in postoperative recovery. Similarly, another study conducted in 2018 reported that the SSI rate after stoma reversal using LSC was 42.5% compared to 7.5% using PSA [14]. The statistical analysis of another study in 2021 also reported PSA as the superior method [15].

There is a lack of locally available data comparing LSC and PSA techniques for stoma reversal. SSIs are a major source of morbidity after stoma reversal, leading to an increased burden on patients and the healthcare systems. In the current era of evidence-based surgical practices, this study will help to determine the better technique for stoma reversal in our local population and generate further research on this topic.

The objective of this study is to compare the frequency of SSI between LSC and PSA of the skin in a stoma reversal procedure.

## Materials and methods

The study was conducted at the Department of General Surgery, Shifa International Hospitals Ltd. (SIH), Islamabad, Pakistan. Permission from the hospital ethics committee and the institutional review board was obtained for the study (approval number: 478-21). This study is a randomized, controlled clinical trial.

The study was conducted between the 14^th^ of March 2021 and the 22^nd^ of November 2022. The sampling technique was non-probability random sampling. The sample size was calculated using the WHO sample size calculator using the hypothesis test for two population proportions (a two-sided test). The confidence interval was 95%. The power of the test (1-β) was 95%, and the level of significance (α) was 5%. The anticipated population proportion 1 (P1) was 42.5%, and the anticipated population proportion 2 (P2) was 7.5%. The minimum sample size in each group was 40 patients. The total sample size was 80 patients.

Patients aged between 20 and 60 years with a BMI between 19 and 35 kg/m^2^ and American Society of Anaesthesiologists (ASA) Class I, II, and III were included. Both colostomy and ileostomy reversal procedures were included in this study. Patients who did not complete the follow-up visits were excluded from the study, as well as patients who had a parastomal hernia in the past and underwent mesh repair, those taking systemic immunosuppressants at the time of surgery, and patients undergoing revision surgery for stoma as well as having end stomas.

A written informed consent was obtained from all the patients admitted to the surgical ward of SIH for elective stoma reversal who met the sample selection criteria. Patients were randomly divided into each group. This process was continued until a sample size of 80 was reached, with 40 in each group. A thorough history and detailed physical examination were performed on all patients. Patients were admitted to the surgical ward through the outpatient department one day before surgery, and gut preparation was done. Baseline investigations, including blood complete picture, urine routine examination, liver function tests, renal function tests, chest radiograph, ECG, and hepatitis B and C screening, were done. The anesthetists did a pre-anesthesia assessment. All patients underwent stoma reversal on the elective list under general anesthesia. A single prophylactic antibiotic dose of intravenous 1 gram ceftriaxone (Rocephin) was administered to all patients during anesthesia induction. Patients were divided into Group A (LSC) and Group B (PSA), containing 40 patients each by randomization. The demographic data of all patients was documented on the proforma.

The incision for stoma takedown was elliptical in Group A and a circumstomal incision in Group B. After adhesiolysis of the stoma, a simple closure or resection and hand-sewn/stapled end-to-end anastomosis were performed. A layer-by-layer linear suturing was done for the fascia of the rectus abdominis muscle. Subcutaneous tissue was sutured by using an absorbable Vicryl 2/0 suture. For skin closure in Group A, the incision was closed by the LSC technique using vertical mattress-interrupted sutures with a non-absorbable Prolene 2/0.

While in Group B, the incision was closed using a purse-string sub-cuticular suture using a Vicryl 2/0 suture. All patients were followed for the development of SSI as per the operational definition on the 7th, 14th, and 28th postoperative days. The result regarding the development of SSI was recorded on the 28th postoperative day. The data of all patients in both groups was recorded on a predesigned proforma. Ten patients did not follow up, so they were excluded as per the exclusion criteria, and the study was continued until a total of 40 patients in each group was obtained.

Data was analyzed by SPSS Statistics version 25.0 (IBM Corp. Released 2017. IBM SPSS Statistics for Windows, Version 25.0. Armonk, NY: IBM Corp.). Descriptive statistics were calculated for both qualitative and quantitative variables. The mean and standard deviation were calculated for quantitative variables like age and BMI. Qualitative variables like gender, ASA class, indication of stoma formation, nature of stoma, and SSI were expressed as frequency and percentages. A chi-square table was constructed and applied to compare the frequency of SSI with the two closure methods. The p-value of ≤0.05 was considered significant.

## Results

The total number of participants in this study was 80, with 40 patients present in each group. The mean age of patients was 44.40±12.77 years. The mean age of patients in Group 1 (LSC) was 43.18±13.44 and in Group 2 (PSA) was 45.63±12.10 years. There were 47 (59%) male and 33 (41%) female cases, with an overall higher male-to-female ratio. In Group 1 (LSC), there were 25 (62.5%) male and 15 (37.5%) female cases. In Group 2 (PSA), there were 22 (55.0%) male and 18 (45.0%) female cases (Table 1).

**Table 1:** Age and gender distribution of two groups LSC: linear skin closure, PSA: purse-string approximation

Group	Mean age (years)	Age range (years)	Male cases (%)	Female cases (%)
Total	44.40±12.77	20-60	47 (59%)	33 (41%)
LSC	43.18±13.44	20-60	25 (62.5%)	15 (37.5%)
PSA	45.63±12.10	23-60	22 (55.0%)	18 (45.0%)

The mean BMI was 24.40±4.15 kg/m^2^. The mean BMI in Group 1 (LSC) was 24.90±4.25 kg/m^2^. The mean BMI in Group 2 (PSA) was 23.89±4.03 kg/m^2^. The nature of the stoma was ileostomy in 61 (76.3%) cases and colostomy in 19 (23.8%) cases. In Group 1 (LSC), the nature of the stoma was ileostomy in 36 (90.0%) and colostomy in four (10.0%) of the cases. In Group 2 (PSA), the nature of the stoma was ileostomy in 25 (62.5%) and colostomy in 15 (37.5%) of the cases. There were 39 (48.8%) patients with ASA Class I, 38 (47.5%) patients with ASA Class II, and three (3.8%) patients with ASA Class III (Table 2).

**Table 2:** BMI, type of stoma, and ASA class of the two groups ASA: American Society of Anesthesiologists, BMI: body mass index, LSC: linear skin closure, PSA: purse-string approximation

Group	Mean BMI (kg/m²)	BMI range (kg/m²)	Ileostomy Cases (%)	Colostomy Cases (%)	ASA Class I (%)	ASA Class II (%)	ASA Class III (%)
Total	24.40±4.15	19.15-34.77	61 (76.3%)	19 (23.8%)	39 (48.8%)	38 (47.5%)	3 (3.8%)
LSC	24.90±4.25	19.15-34.77	36 (90.0%)	4 (10.0%)	18 (45.0%)	21 (52.5%)	1 (2.5%)
PSA	23.89±4.03	19.26-33.12	25 (62.5%)	15 (37.5%)	21 (52.5%)	17 (42.5%)	2 (5.0%)

The indication of stoma formation was bowel obstruction in five (6.3%) patients, bowel perforation in 12 (15.0%) patients, diversion for benign disease in 29 (36.3%) patients, and diversion for malignant disease in 34 (42.5%) patients. In Group 1 (LSC), the indication of stoma formation was bowel obstruction in two (5.0%) patients, bowel perforation in 11 (27.5%) patients, diversion for benign disease in nine (22.5%) patients, and diversion for malignant disease in 18 (45.0%) patients. In Group 2 (PSA), the indication of stoma formation was bowel obstruction in three (7.5%) patients, bowel perforation in one (2.5%) patient, diversion for benign disease in 20 (50.0%) patients, and diversion for malignant disease in 16 (40.0%) patients (Table 3).

**Table 3:** Reasons for stoma formation LSC: linear skin closure, PSA: purse-string approximation

Group	Bowel obstruction (%)	Bowel perforation (%)	Diversion for benign disease (%)	Diversion for malignant disease (%)
Total	5 (6.3%)	12 (15.0%)	29 (36.3%)	34 (42.5%)
LSC	2 (5.0%)	11 (27.5%)	9 (22.5%)	18 (45.0%)
PSA	3 (7.5%)	1 (2.5%)	20 (50.0%)	16 (40.0%)

The overall frequency of SSI in all the patients was 18/80 (22.5%). The frequency of SSI in Group 1 (LSC) was 6/40 (15.0%), and in Group 2 (PSA) it was 12/40 (30.0%). The frequency of SSI in Group 2 (PSA) was twice as high as in Group 1 (LSC); however, the p-value was calculated to be 0.108. Therefore, this difference was statistically insignificant (Table 4).

**Table 4:** Frequency of SSI SSI: surgical site infection, LSC: linear skin closure, PSA: purse-string approximation

Group	Frequency of SSI (%)	p-value
Total	18/80 (22.5%)	0.108
LSC	6/40 (15.0%)	
PSA	12/40 (30.0%)	

Stratification for age, BMI, and ASA class showed no effect modification on the frequency of SSI among the two groups. Stratification for gender revealed effect modification. Among the male patients, there was a higher frequency of SSI in Group 2 (PSA) as compared to Group 1 (LSC), which was statistically significant (p-value=0.049). However, among female patients, there was no statistically significant difference between the two groups (p-value=0.876). Stratification for the nature of the stoma showed no effect modification on the frequency of SSI among the two groups. SSI was present in 10/61 ileostomy reversals (16.4%) and in 8/19 colostomy reversals (42.1%). Stratification for the indication of stoma formation showed no effect modification on the frequency of SSI among the two groups (Table 5).

**Table 5:** Stratification of variables against SSI SSI: surgical site infection, LSC: linear skin closure, PSA: purse-string approximation, BMI: body mass index

Stratification	Group	SSI	No SSI	p-value
Age groups (20-40 years)	LSC	3	13	0.414
Age groups (20-40 years)	PSA	5	11	0.414
Age groups (41-60 years)	LSC	3	21	0.155
Age groups (41-60 years)	PSA	7	17	0.155
BMI (19.00-25.00 kg/m^2^)	LSC	4	18	0.627
BMI (19.00-25.00 kg/m^2^)	PSA	6	19	0.627
BMI (25.01-35.00 kg/m^2^)	LSC	2	16	0.054
BMI (25.01-35.00 kg/m^2^)	PSA	6	9	0.054
Gender (male)	LSC	3	22	0.049
Gender (male)	PSA	8	14	0.049
Gender (female)	LSC	3	12	0.876
Gender (female)	PSA	4	14	0.876
ASA Class (ASA 1)	LSC	2	16	0.101
ASA Class (ASA 1)	PSA	7	14	0.101
ASA Class (ASA ≥2)	LSC	4	18	0.530
Nature of stoma (ileostomy)	LSC	4	32	0.181
Nature of stoma (ileostomy)	PSA	6	19	
Nature of stoma (colostomy)	LSC	2	2	0.719
Nature of stoma (colostomy)	PSA	6	9	
Indication of stoma formation (bowel obstruction)	LSC	0	2	0.361
Indication of stoma formation (bowel obstruction)	PSA	1	2	
Indication of stoma formation (bowel perforation)	LSC	4	7	0.460
Indication of stoma formation (bowel perforation)	PSA	0	1	
Indication of stoma formation (diversion for benign disease)	LSC	1	8	0.183
Indication of stoma formation (diversion for benign disease)	PSA	7	13	
Indication of stoma formation (diversion for malignant disease)	LSC	1	17	0.110
Indication of stoma formation (diversion for malignant disease)	PSA	4	12	

## Discussion

The most prevalent complication following stoma reversal is SSI. SSIs constitute a significant source of morbidity, leading to prolonged hospitalization, increased financial burdens, and detrimental impacts on the patient's quality of life. Alexander et al. estimated the cost associated with a single SSI following colonic surgery to be approximately $2,600 [16]. Consequently, various techniques for wound closure have been documented in the literature to address this issue. One relatively innovative approach is the PSA method, which has demonstrated a reduction in the SSI rate compared to the conventional LSC method [17]. While some studies have reported significant reductions in the SSI rate, others have failed to replicate this advantage.

Sajid et al. conducted a meta-analysis of randomized controlled trials (RCTs) comparing PSA to LSC for stoma reversal procedures [18]. Three RCTs, encompassing 206 patients in total, were included in their analysis. Of these, 105 patients were assigned to the PSA group, while 101 were assigned to the LSC group. The trials exhibited no heterogeneity. The incidence of SSIs in the PSA group was a mere 2%, in stark contrast to the 32.6% observed in the LSC group (p-value<0.0001). However, it should be noted that this meta-analysis solely incorporated RCTs that focused on ileostomy reversals, excluding colostomy reversals, which could potentially introduce bias.

In contrast, our study encompassed both ileostomy and colostomy reversals (61 ileostomy cases and 19 colostomy cases). Furthermore, all three RCTs had relatively small sample sizes, totaling 206 patients across all trials. Hence, the authors concluded that further validation through multicenter RCTs would be imperative.

Our study showed that age is unrelated to wound infection after stoma closure, whether LSC or PSA. This has been the case in other studies as well. However, HbA1c levels and obesity have been closely linked with SSI. Liang et al. in 2013 showed that patients with SSI were likely to have high HbA1c levels (6.5% vs. 5.9%, p-value=0.02) [19]. Our study did not include the data for HbA1c, but BMI was stratified against SSI.

Our study showed that the greater the BMI, the greater the chances of SSI, irrespective of LSC or PSA. However, the p-value was not significant. This hasn't been the case in the literature, and various studies have affirmed the correlation between BMI and SSI in the case of ileostomy wound closure. Mirbagheri et al. showed in their research that obesity was one of the main reasons causing wound infection post-stoma closure (p-value=0.02) [20]. Similar results were obtained by Hourigan et al. in 2011, ranking obesity as one of the leading causes of wound infection in stoma closure [21]. Chun et al. reviewed 123 patients and observed a 64.2% overall complication rate in patients with a BMI>30, predicting a higher risk for infection [22].

In our study, the ASA class was stratified against SSI, and no significant correlation was found between the two. In the cases of LSC and PSA, the p-value was insignificant for the ASA class. However, few studies have shown that with ASA Class III-IV, there is a greater chance of wound infection with stoma closure [23].

The type of stoma, ileostomy or colostomy, affects the rate of SSI in cases of stoma closure. Our study did not find a significant difference between the rates of SSI among the two. Literature has shown that this type of stoma occasionally affects the SSI rate, which in turn impacts the patient's quality of life and prolongs hospital stays. Studies have shown that an end colostomy is more likely to cause infection. This is because the colon, particularly the descending colon, harbors a higher bacterial count and is associated with an increased risk of SSI [24,25].

An indication of a stoma is believed to play an important role in SSI. In our study, p-values for bowel obstruction and perforation were insignificant. This trend, however, has not been consistent, and literature shows that stoma formation in cases of emergency surgery usually has a propensity toward infection upon closure [26-28].

Sureshkumar et al. conducted a prospective RCT spanning two years. The findings indicated that the incidence of SSI in patients undergoing stoma reversal via the LSC technique was 42.5%, as opposed to 7.5% in the PSA group (p-value=0.003) [14]. Similarly, Ali et al. observed that the incidence of SSI in patients undergoing stoma reversal via the LSC technique was 22.85%, whereas it was 5.41% in those undergoing the PSA technique (p-value=0.023) [15]. In our study, the p-value remained insignificant for LSC and PSA throughout. In the case of an ileostomy closed by LSC and PSA techniques, the p-value was 0.181, while for a colostomy, it was 0.719.

The STOMA trial by O'Leary et al. also aimed to compare the two closure modalities [29]. The researchers found that the rate of SSI was significantly higher in the LSC group (30%) compared to the PSA group (8%) (p-value=0.03).

Conversely, in an RCT conducted by Amano et al. comparing PSA with LSC with a drain, it was reported that there was no statistically significant difference (p-value=0.35) between the two groups regarding the incidence of SSI, with frequencies of 8.9% in the LSC group and 5.0% in the PSA group [30]. However, it should be noted that in their study, the surgeons performing the PSA had varying levels of expertise and received guidance from experienced colleagues. This variation in the surgeons' expertise may introduce potential bias into the study.

In our study, the overall incidence of SSI was 18 out of 80 patients (22.5%). Specifically, the SSI rate in the LSC group was six out of 40 patients (15.0%), while 12 out of 40 patients (30%) were in the PSA group. However, this difference did not attain statistical significance (p-value=0.108).

Our study had the limitations of being a single-center study. The sample size in each group is 40, and this sample size is not enough to make conclusive remarks. We performed stratification to account for effect modifiers. No differences were observed in age, BMI, ASA class, nature of stoma, and the indication for stoma formation upon stratification. However, gender-based stratification revealed a higher incidence of SSI in the PSA group compared to the LSC group among male patients, which was statistically significant (p-value=0.049).

## Conclusions

While PSA has exhibited promise in reducing SSI rates and enhancing aesthetic outcomes and patient satisfaction, there is still enough data favoring LSC. Moreover, insufficient data is available for our population to make a definitive statement. Consequently, further research on this topic is warranted, preferably involving larger sample sizes and multicenter RCTs, to establish which technique is superior in SSI reduction.
